# Reduced Transition Temperature in Al:ZnO/VO_2_ Based Multi-Layered Device for low Powered Smart Window Application

**DOI:** 10.1038/s41598-020-58698-w

**Published:** 2020-02-04

**Authors:** Makhes K. Behera, Leslie C. Williams, Sangram K. Pradhan, Messaoud Bahoura

**Affiliations:** 1 Center for Materials Research, Norfolk State University, Norfolk, VA 23504 United States; 2 Norfolk State University, Norfolk, VA 23504 United States

**Keywords:** Energy science and technology, Materials science, Nanoscience and technology

## Abstract

The metal-to-insulator transition (MIT) closest to room temperature of 68–70 °C as shown by vanadium oxide (VO_2_), compared with other transition metal oxides, makes it a potential candidate for smart window coating. We have successfully fabricated a potential smart window device after the optimum design of a multilayered thin film structure made out of transparent conducting oxide (aluminum doped zinc oxide) and pure VO_2_ using pulsed laser deposition technique. This comprehensive study is based on two different configurations for multi-layered structure approach, with the intention to reduce the transition temperature, as well as to maintain the MIT properties that would strengthen the potential of the structure to be used for a smart window device. By creating a multi-layered structure, we were able to create a low powered device that can operate less than 15 V that leads to significant decline in the infrared transmission by a magnitude of over 40% and provided sufficient heat to trigger the MIT at a temperature around 60 °C, which is almost 10 °C lower than its bulk counterpart. This finding would positively impact the research on VO_2_ thin films, not only as smart windows but also for numerous other applications like bolometers, infrared detectors, Mott transistors and many more.

## Introduction

Energy in today’s world is the most crucial problem humanity is going to face by the year 2050. The increasing population, depleting natural resources and unavailability of sufficient energy generated from renewable energy sources has led to a dramatic rise in energy prices. While researchers all around the globe have pooled their efforts to find alternate sources of energy and better energy storage solutions, energy saving is another approach that researchers have focused on by developing low powered devices for practical applications. One of the major energy consuming applications has been the HVAC (heating, ventilation and air conditioning) system in residential and commercial buildings, consuming approximately 46% of the total used energy. This calls for an energy saving solution. One possible approach that has been discussed quite often in the research community, is the use of smart windows. Smart windows are, essentially, coated windows which will be able to modulate the temperature of the room depending on the surrounding environment.

The concept of MIT was first discovered in the year 1959 by F. J. Morin^1^ when he studied different transition metal oxides. It refers to the change in a material’s properties from being an insulator to being a metal after exposing them to a critical temperature, generally referred to as the transition temperature (T_c_). While the property of MIT is exhibited by several transition metal oxides such as VO, Ti_2_O_3_, V_2_O_3_ and VO_2_, VO_2_ has showed the material of greatest interest owing to its transition temperature around ~68–70 °C, making it the closest to RT among all the other metal oxides (T_c_ for V_2_O_3_ ~ −108 °C, Ti_2_O_3_ ~ 127 °C −227 °C, VO ~ −147 °C)^1^. Further research into VO_2_ showed that after reducing the dimension (thin film), the transition temperature in VO_2_ can be tuned to be even closer to room temperature (RT), getting it as low as ~27 °C. The film’s transition temperature can be tuned depending on the thickness, strain, stoichiometry, growth parameters and growth techniques of the films^2–12^. Furthermore, doping the films with high valence element such as W, Mo or negatively charged ions such as F would bring down the transition temperature close to RT with a doping percentage even as low as 6%. It was also observed that a similar transition was likewise seen in the optical properties of the films in the near infra-red (NIR) region. This means that when VO_2_ undergoes a MIT, it changes the structure from being a monoclinic, electrically insulating IR transparent state, to a tetragonal, electrically conducting IR blocking state^13,14^, which makes it unique to be used as a smart window coating. Besides smart windows, VO_2_ could potentially also be used in applications such as uncooled bolometers, Mott-transistors, IR detectors, laser protection, metamaterials and several other applications^3,14–16^.

However, despite the recent breakthroughs in doping and growth techniques, VO_2_ thin films still have not been able to make their way to the commercial market. Several issues play a role in it, first of which being the difficulty in growing high quality thin films of VO_2_. Vanadium, being a transition metal can exhibit several oxidation states starting from +2 all the way up to +5. It has been found that vanadium can exhibit as many as 16 different oxide states, with notable ones being VO, V_2_O_3_, VO_2_ and V_2_O_5_. VO_2_ is a meta-stable oxide of vanadium which has a tendency to form the more stable V_2_O_5_, which doesn’t exhibit the same MIT as VO_2_^17,18^. Therefore, obtaining VO_2_ phase in the right stoichiometry exhibiting a good MIT property has remained a difficult task. Several growth techniques have been adopted to grow VO_2_ thin films such as RF/DC magnetron sputtering, chemical vapor deposition (CVD), atomic layer deposition (ALD), pulsed laser deposition (PLD) and several hydrothermal routes. Techniques using a low substrate temperature, such as the hydrothermal, CVD and ALD have not been able to grow high quality epitaxial VO_2_ thin films. However, molecular beam epitaxy (MBE) and sputtering are capable of growing epitaxial films, but with significant degradation to the MIT properties. Nevertheless, PLD stands out as technique of choice because of the ability to control parameters such as substrate temperature and oxygen partial pressure precisely, which makes it suitable to grow a metastable material such as VO_2_^19–29^.

Another problem, associated with even the most recent advancements, includes the MIT properties of the film itself. A typical MIT curve has not only the transition temperature but a few other parameters determining the quality of MIT, such as the sharpness, magnitude and the hysteresis width of the curve. Ideally, for an application in smart window coating, an MIT with a high magnitude (for maximum IR blocking)^30,31^, high sharpness (for fast IR transparent to IR blocking state) and a narrow hysteresis width (for stable operation) is desirable. However, it has been found that, using several methods, while lowering the transition temperature this is accompanied with a degradation in other MIT parameters which makes it unsuitable to be used frequently for certain applications. Another issue that exists with VO_2_, for practical applications, lies in its optical properties, having a low visible range transmittance (~40–45%).

In this paper, we report a reduction in the transition temperature of the VO_2_ thin films with a marginal degradation to the other MIT properties. Furthermore, we report a novel structural approach of multi-layered device using Al:ZnO thin films as a resistive heater layer to provide the additional temperature needed to trigger the MIT by applying a very low voltage^32,33^.

## Results and Discussion

**Figure 1:**
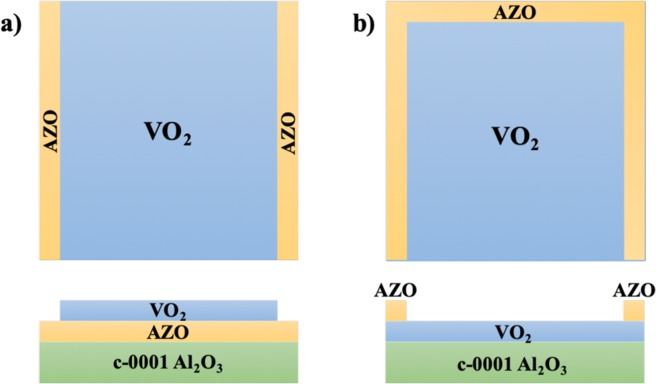
(**a**) S-1 configuration of the sample with a VO_2_/AZO multi-layered structure with the exposed AZO layer to be used for supplying the voltage to the films. (**b**) S-2 configuration of the sample with an AZO/VO_2_ multi-layered structure with the AZO strip heater layer at the edges to be used for supplying the voltage to the films.

The multi-layered structures used for demonstration of a potential smart window device are as shown in Fig. 1(a,b). While the structure S-1 in Fig. 1(a) follows a structure with the AZO layer at the bottom to heat the VO_2_ films, S-2 structure in Fig. 1(b) has the AZO layer deposited along the edges to heat the films from the top.

**Figure 2:**
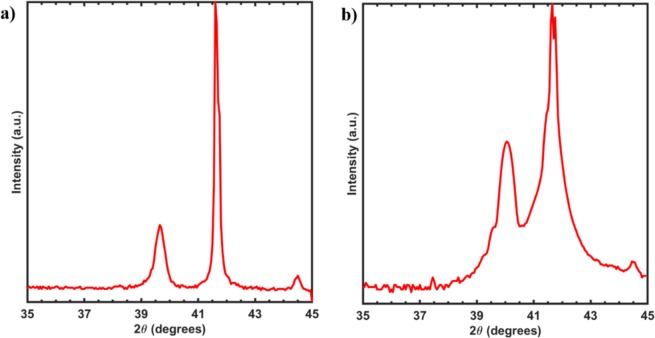
(**a**) X-ray diffraction patterns for the non-annealed as-deposited VO_2_ thin film with the VO_2_ peak on the film seen at a 2θ angle of 39.7 degrees (**b**) X-ray diffraction patterns for the VO_2_ thin film annealed at 660 °C with the VO_2_ peak on the film seen at a 2θ angle of 40.1 degrees corresponding to the monoclinic structure of VO_2_ (002).

Figure 2(a,b) show the X-Ray diffraction pattern for the 50 nm VO_2_ thin films before and after annealing. It was observed from the graph that the (020) peak of VO_2_ was shifted towards a higher 2θ angle upon annealing indicating a possible increase in the oxygen concentration in the films caused by filling up of the oxygen vacancies in the films upon annealing^34–36^.

**Figure 3:**
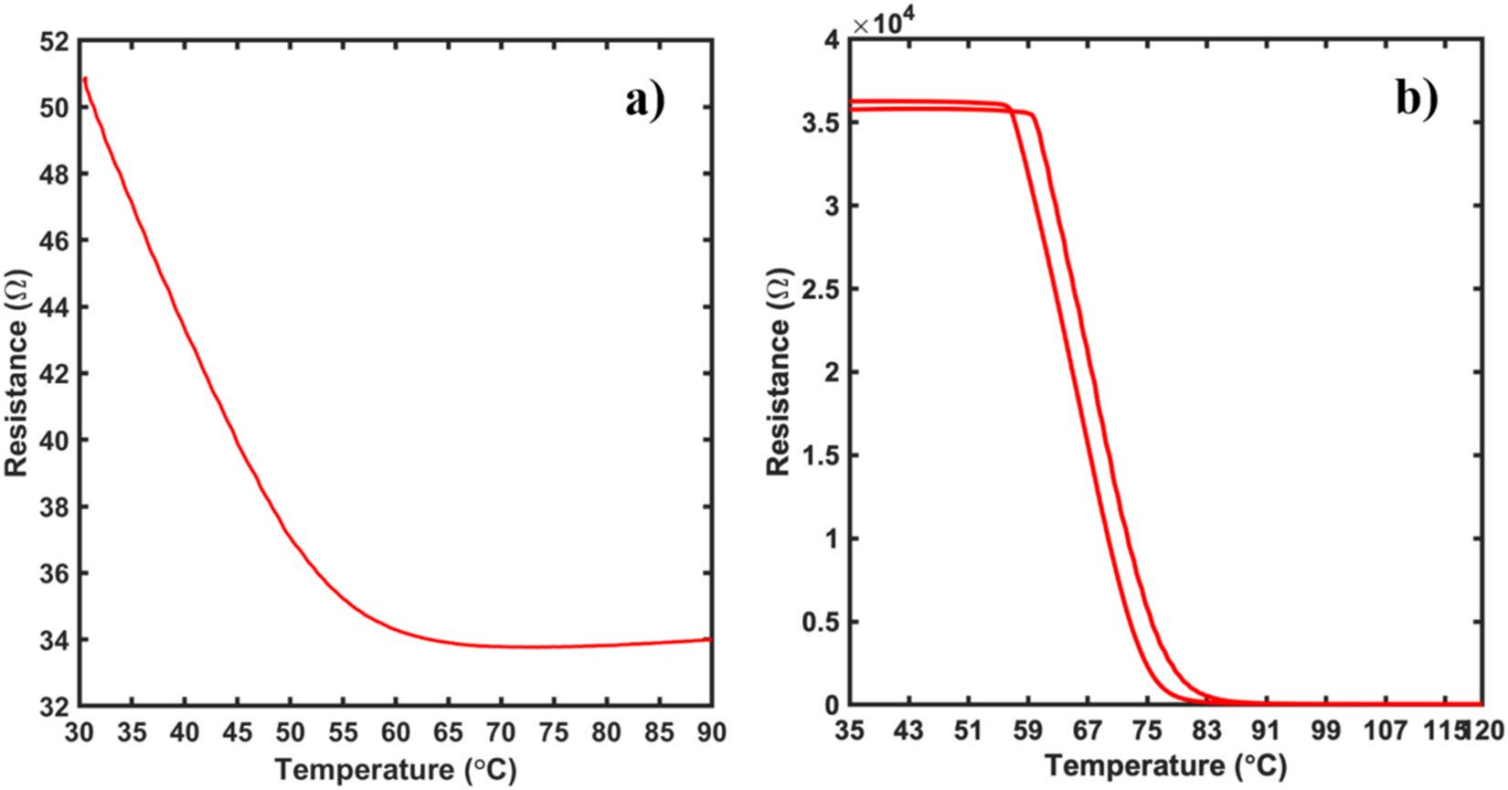
Temperature dependent resistance measurement for (**a**) Non-Annealed and (**b**) Annealed sample in a linear four-point probe configuration. As seen clearly, there is a dramatic increase in the resistance of the films by about 700 times. Furthermore, while the non-annealed sample showed a semiconducting behavior, the annealed sample demonstrated a metal-to-insulator transition indicating a stoichiometry close to VO_2_.

**Figure 4:**
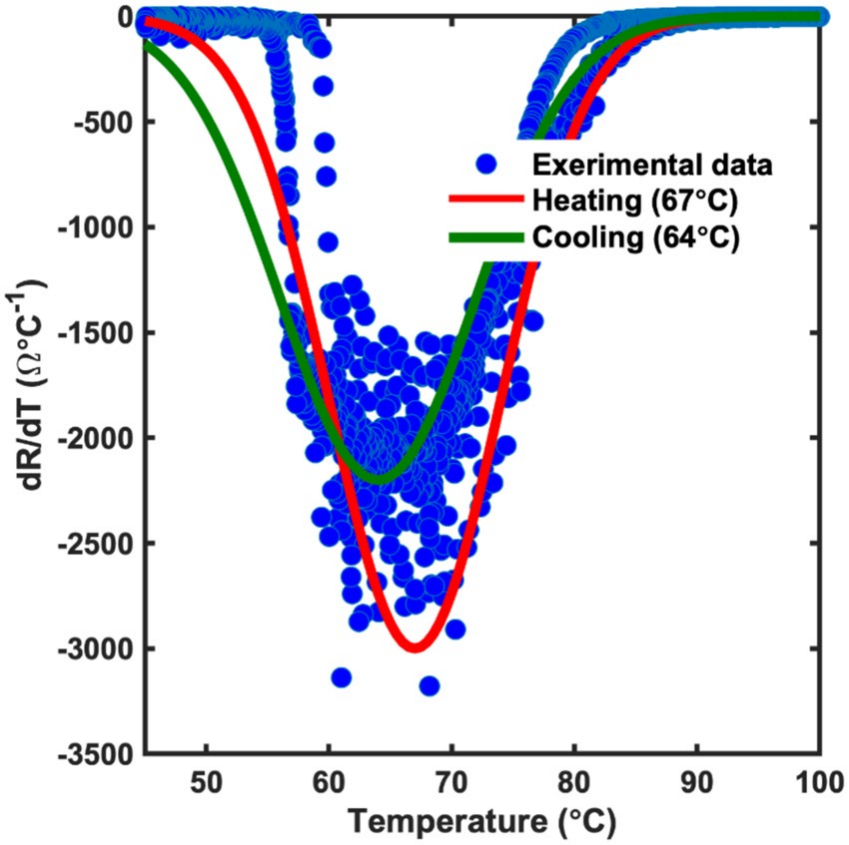
Determination of the transition temperature in VO_2_ thin films. The transition temperature of the films was determined by the analysis of the temperature dependent slope plot of the films calculated from the data obtained from the linear four probe measurement. The experimental data was fitted to a gaussian profile in order to obtain the transition temperature during heating as well as cooling. As seen from the figure, the transition temperature of the film was found to be ~67 °C with a hysteresis width of 3 °C.

A similar effect was seen in the electrical properties of the films as we saw the resistance of the films increase by 1000 times and the change of the electrical properties demonstrate a semiconducting behavior having a metal-insulator transition phase. The electrical behavior of the films is shown in Fig. 3(a,b). The annealed film displaying a MIT property was found to have a transition temperature of ~67 °C, similar to the theoretical MIT temperature seen in bulk VO_2_ thin films. This could be due to the filling up of oxygen vacancies that were formed prior to annealing. Annealing the films under a higher oxygen partial pressure environment brought the film stoichiometry closer to the desirable ratio for the films to display a metal-to-insulator transition^37–41^. The transition temperature of the films was calculated by using the plot of the temperature differential of the resistance with respect to temperature. As seen from the plot in Fig. 4, the films were found to have a transition temperature of 67 °C with a hysteresis of 3 °C between the heating and cooling curve.

**Figure 5:**
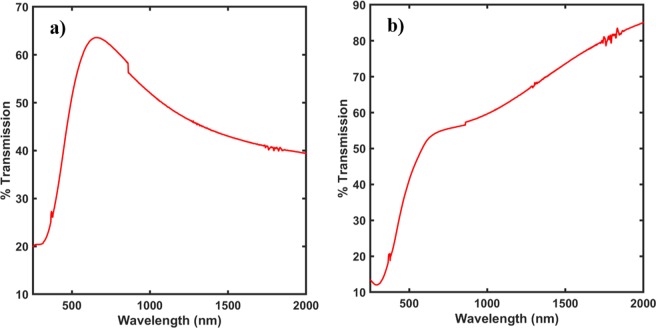
UV/Vis transmission spectra from a wavelength range of 250 nm to 2000 nm for (**a**) Non-annealed sample (**b**) Annealed sample. As seen from the curves, the transmission in the IR region increases for the annealed sample while the visible range transmission stays almost the same.

Figure 5(a,b) show the optical properties of the films, measured using the UV/Vis spectrometer. As it can be clearly seen from the plots that the transmission in the visible wavelength spectrum changed by 5% while the transmission was taken near IR region, but at 2000 nm range, it increased by approximately 30% at room temperature.

The changes in the electrical and optical properties of the films were due to the change in the oxygen concentration of the films after annealing in an oxygen rich atmosphere which help convert the films from being in an oxygen lacking state to a monoclinic VO_2_ phase that resulted in the films establishing a MIT as well as optical properties similar to bulk VO_2_ films.

**Figure 6:**
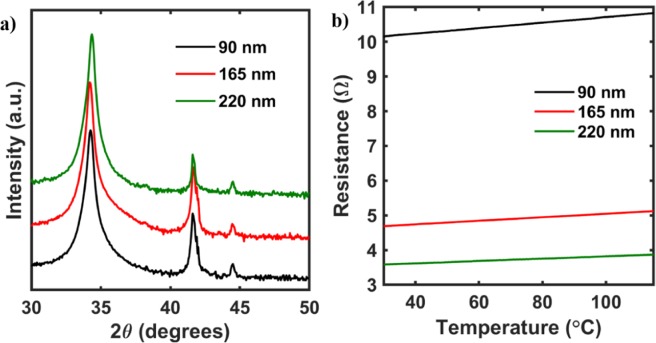
(**a**) X-ray diffraction patterns for the AZO thin film with the AZO peak on the film seen at a 2θ angle of 32.5 degrees. (**b**) Electrical properties of the AZO thin films grown at 350 °C plotted as a function of temperature. As seen from the electrical data, the resistance of the thin films increases with an increase in temperature suggesting an Ohmic behavior. Furthermore, the resistance of the films decreases with an increase in thickness of the films.

Figure 6(a) shows the X-Ray diffraction patterns for the AZO films of different thickness 90 nm, 165 nm and 220 nm. The presence of distinct (002) peak at ~32.5 degrees is the characteristic feature of ZnO also reported earlier^32,33^. All the as-grown films were then characterized using the linear 4 probe technique in order to determine the material behavior, as shown in Fig. 6(b). All the films show an Ohmic behavior with the resistance of the films increasing linearly with the increase in temperature. Furthermore, the resistance of the films, at room temperature, is reduced with an increase in the thickness of the films. This property could be due to an increase in the oxygen vacancy concentration of the films with an increase in the film thickness, which is due to the deposition of the films in an oxygen deficient atmosphere.

**Figure 7:**
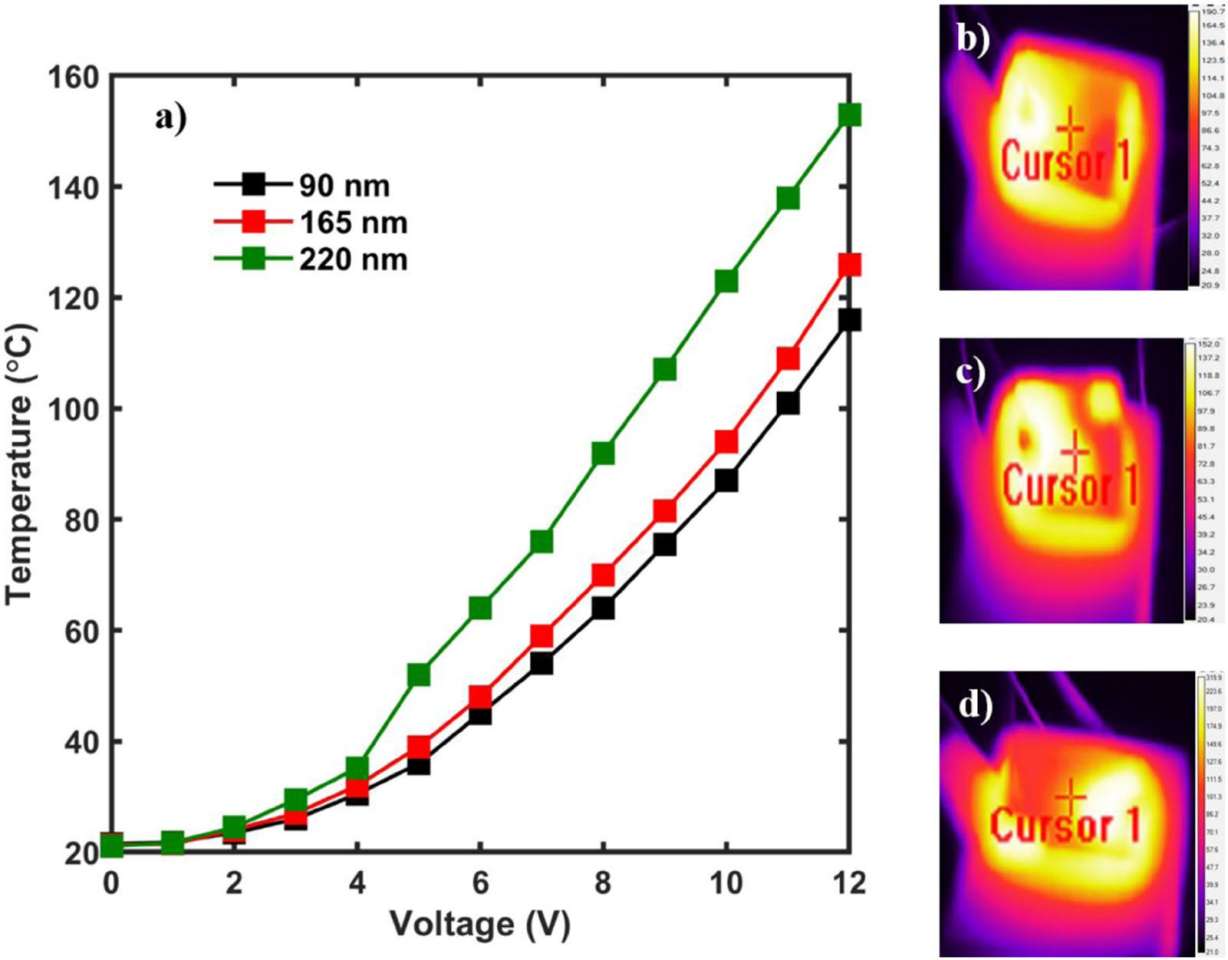
(**a**) Electro-thermal measurements on the Al doped Zinc oxide thin films for different thickness. As seen from the plot, the properties improve with an increase in thickness from 90 nm to 220 nm. (**b–d**) Thermal images of the AZO films upon application of 12 V for thickness of 90 nm, 165 nm and 220 nm, respectively. As seen from the images, the heating is fairly uniform across the film surface as measured by the IR camera.

As deposited AZO films demonstrated a Joule heating effect through an external bias voltage and temperature was monitored using an IR camera. It was seen that the heating effect of the films improved with an increase in layer thickness from 90 nm to 220 nm, as shown in Fig. 7(a). The enhancement in the heating effect could be attributed to the reduction in resistance of the films with higher thickness. The thermal images of all the films having thickness of 90 nm, 165 nm and 220 nm measured at ~12 V are shown in Fig. 7(b–d) respectively.

**Figure 8:**
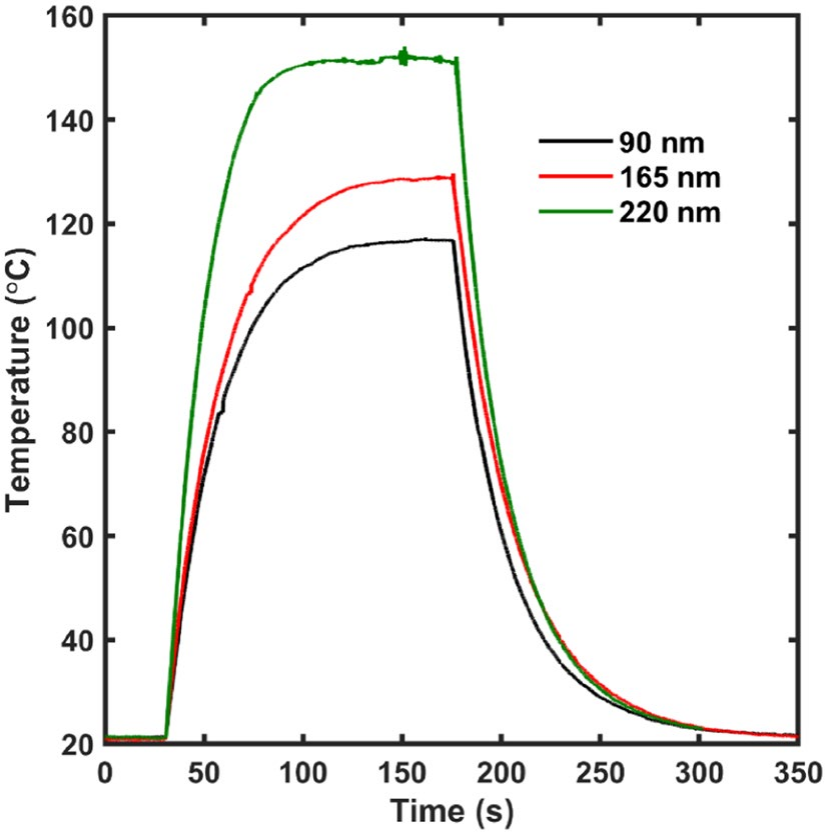
Heating profile information for the Al doped zinc oxide thin films upon application of 12 V. As seen from the profile, the films are able to heat up to the maximum temperature at a faster rate and stays at the constant temperature indicating a good electro-thermal performance.

The application of a direct voltage of 12 V to the films showed reproducible results and good heating/cooling profiles. The films show a sharp rise and fall in temperature as voltage is applied without any noticeable spikes in temperature. Furthermore, as seen from the IR images of the films in Fig. 7(b–d), the heating characteristic of the films is uniform in nature which makes AZO thin film a good candidate as the intermediate heating layer for the MIT effect of VO_2_ thin films. The heating profile of the different thickness films with applied voltage of 12 V is shown in Fig. 8.

**Figure 9:**
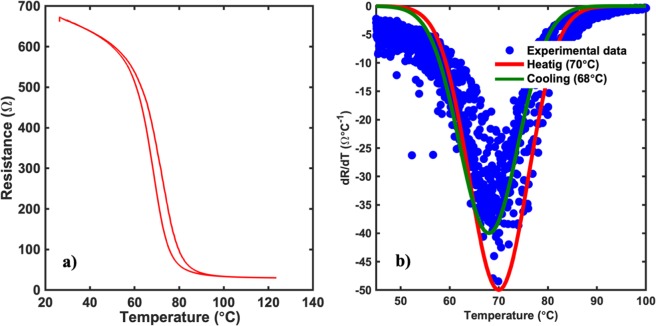
(**a**) Electrical characterization of the VO_2_/AZO thin film structure in S-1 configuration using the linear four-point probe setup at room temperature. As seen, the resistance of the films at RT reduce dramatically causing an overall reduction to the magnitude of the MIT in the films. (**b**) Temperature dependent slope profile of the experimental data obtained from the linear four probe measurement. As seen from the figure, the films have a transition temperature of ~70 °C with a hysteresis width of 2 °C.

Based on the results obtained from pristine AZO and VO_2_ thin films, 165 nm of AZO thin film was used as the intermediate layer with VO_2_ film deposited on top of it using the S-1 configuration, leaving parts of AZO exposed on both ends for external bias. The electrical properties of the VO_2_ films, were able to demonstrate a metal-to-insulator transition similar to the pristine films. The MIT shown in the S-1 configuration was found to have a transition temperature of 70 °C comparable to the pristine VO_2_ films, which showed a transition temperature of ~67 °C, as mentioned before. Furthermore, it was found that, although the sharpness of the transition did not change by much, the magnitude of transition was reduced drastically as much as 50 times, owing to the dramatic reduction in the room temperature resistance of the VO_2_ thin films when grown over the AZO thin film. This change could be caused by the highly conductive nature of the AZO film. The electrical measurement on the film using the linear four-point probe configuration is shown in Fig. 9(a). A similar method was used to determine the transition temperature of the multi-layer film in S-1 configuration as the pristine one. The plot used to determine the transition temperature of the film is shown in Fig. 9(b). As seen from the figure, the films had a transition temperature of 70 °C with a hysteresis of 2 °C between the heating and cooling curve.

However, it was also found that AZO thin films no longer displayed a Joule heating effect when a voltage was applied to them. This could be due the vast differences in the growth conditions between the AZO and the VO_2_ films, namely the substrate temperature and the oxygen partial pressure. While the AZO film was grown at a lower substrate temperature of 350 °C with no oxygen partial pressure, the VO_2_ films deposited on top of the AZO layer were grown at a higher substrate temperature of 630 °C with oxygen partial pressures of 1 mTorr and 100 mTorr during deposition and annealing, respectively. We think that the presence of oxygen and the higher temperature exposure of the AZO layer during the deposition of VO_2_ thin film, might be responsible for the filling up of the oxygen vacancies in the films and resulting in the vanishing of the Joule heating effect.

The loss of the Joule heating effect and the dramatic reduction in the magnitude of the MIT of the VO_2_ thin films defeated the goal of having a low powered smart window device despite being able to reduce the transition temperature. However, changing the structural design of the device could induce the same strain-based reduction in the MIT temperature, while still maintaining the heating effect of the AZO films and the magnitude of the MIT. The films grown in the S-2 multi-layered configuration fulfil these conditions. The films were grown in an AZO/VO_2_ configuration as opposed to the VO_2_/AZO in the S-1 structure, while masking off part of the VO_2_ film so that the AZO films can be deposited on the edges in a strip structure to supply enough heat to the VO_2_ thin films without affecting its properties. Since AZO films are grown at a much lower substrate temperature, without using any oxygen partial pressure, the effect of depositing AZO on the VO_2_ thin films should be minimal.

**Figure 10:**
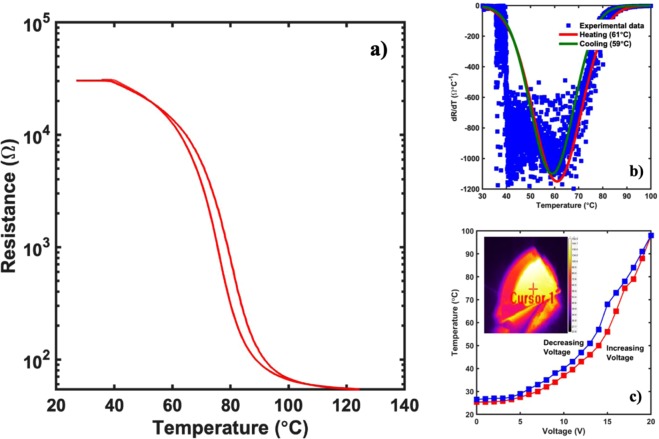
(**a**) Electrical characterization of the AZO/VO_2_ thin film structure in S-2 configuration using the linear four-point probe setup. As seen, the resistance of the films at RT does not change as compared to the VO_2_ thin films by themselves. (**b**) Temperature dependent slope profile of the experimental data obtained from the linear four probe measurement. As seen from the figure, the films have a transition temperature of ~61 °C with a hysteresis width of 2 °C. (**c**) Electro-thermal data on the AZO/VO_2_ films in S-2 configuration with the thermal image of the film in the inset. As seen, there is a sharp increase in the temperature between 9 V and 10 V indicating a transition from insulating phase to a metallic phase, thereby improving the electro-thermal properties as well.

**Figure 11:**
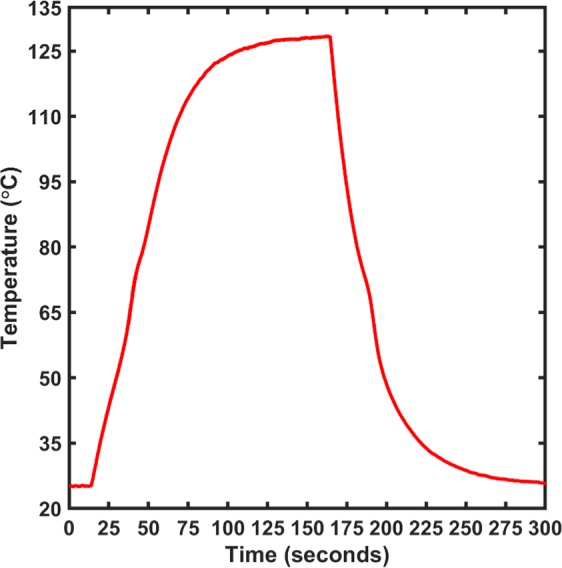
Heating profile information for the AZO/VO_2_ thin films in S-2 configuration upon application of 12 V. As seen from the profile, the films are able to heat up to the maximum temperature in approximately 75 seconds and stays at the constant temperature indicating a good electro-thermal performance.

As per the prediction, the multi-layered films grown in the S-2 configuration brought the magnitude of the MIT back to the one seen in the as deposited VO_2_ thin films while reducing the transition temperature and maintaining the Joule heating effect in the AZO layer. The electrical properties of the VO_2_ thin films measured using linear four probe setup as shown in Fig. 10(a). The plot used to determine the transition temperature of the film using a similar analysis as used for the pristine and multi-layered films in the S-1 configuration as shown in Fig. 10(b). The films had a reduced transition temperature of 61 °C with a hysteresis of 2 °C between the heating and the cooling curve. As mentioned earlier, the transition temperature of the VO_2_ thin films depend on the strain on the films. The addition of the AZO layer on to the VO_2_ films might be induced additional strain on the films which causes the reduction of the transition temperature in VO_2_ films. Furthermore, the electro-thermal properties of the films were measured using the IR camera and the temperature profile with respect to the applied voltage is shown in Fig. 10(c). As seen clearly from the figure, there is a sharp rise in the temperature after 12 V from 43 °C to 98 °C, with a temperature difference of about 55 °C. This trend in the temperature-voltage profile could be attributed to the MIT of the films as all the temperatures were measured on the VO_2_ surface of the multi-layered structure (inset of Fig. 10(c)). The initial slower increase in the temperature of the films upon application of voltage could be attributed to the joule heating effect in the AZO films. As the films reach the transition temperature of the VO_2_ films, the change in the VO_2_ film property to a metallic behavior causes a sharper increase in the film temperature. The sudden increase in temperature of the multi-layered films upon application of the initial heat due to the AZO thin film layer can be explained by the variation of the thermal conductivity in the VO_2_ films across the metal to insulator transition. As reported by Oh *et al*., an increase in the thermal conductivity, very similar to the increase in the electrical conductivity of the films during the MIT was seen^42^. The transition of VO_2_ due to the heat generated by AZO film above 12 V is enough to trigger the MIT transition in the films, converting them from being in a poor heat conducting insulating state to being in a high heat conducting metallic state, bringing about the sudden rise in temperature. Also, as seen from the IR image in the inset of Fig. 10(c), the AZO part of the film is at a considerably lower temperature as compared to the VO_2_ part of the film at 12 V, which heated up uniformly and rapidly. The temperature profile for the multi-layered film upon direct application of 12 V to the film is shown in Fig. 11. As seen from the figure, the temperature of the VO_2_ films increased at a slower rate until it reached 60 °C, after which the MIT was triggered in the films resulting in a sharp rise in temperature.

**Figure 12:**
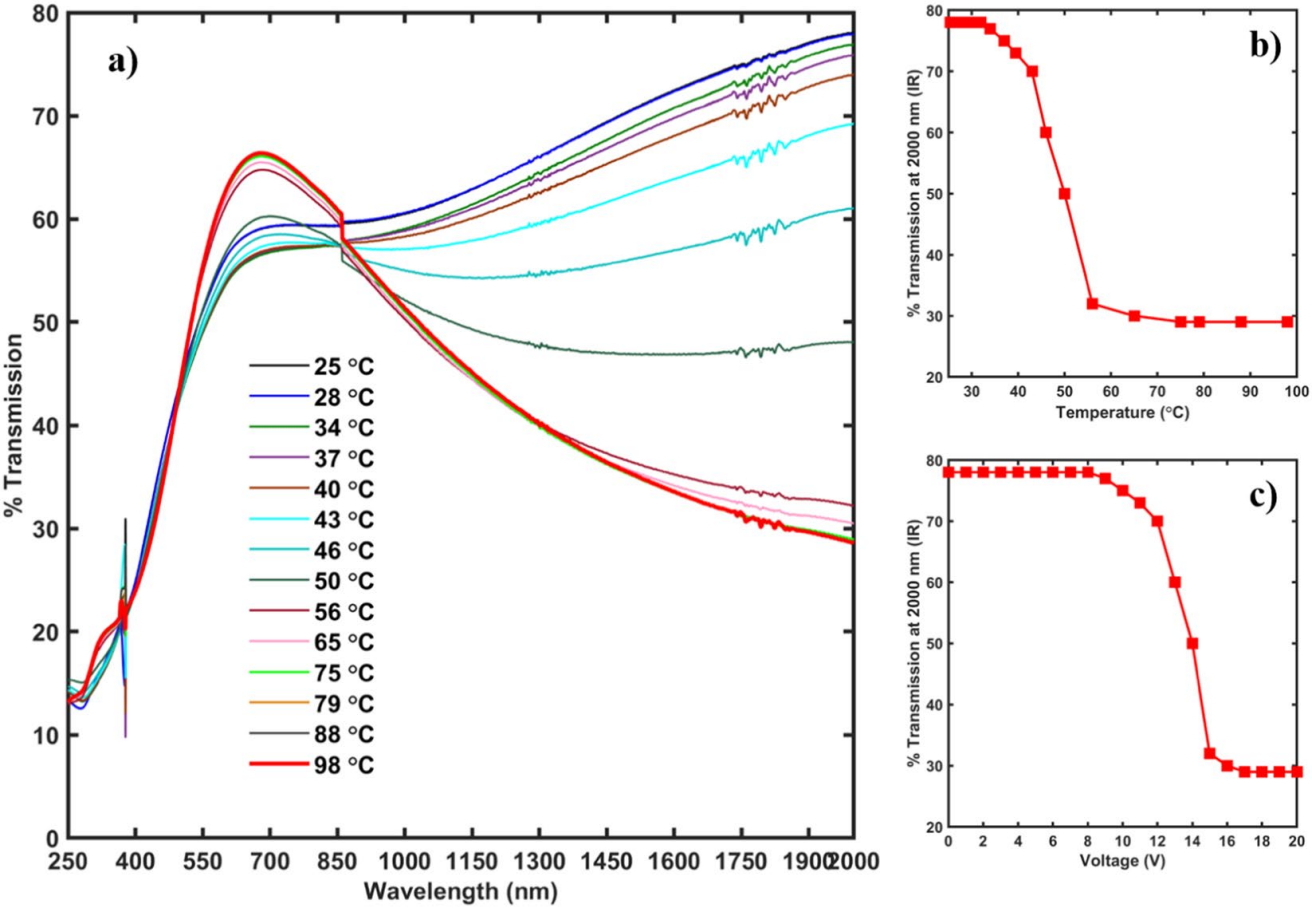
(**a**) Temperature UV/Vis transmission spectra for AZO/VO_2_ thin films in S-2 configuration. As seen from the figure, there is a significant reduction in the IR transmission with the increase in temperature demonstrating the potential of the multi-layered films in S-2 configuration for smart window coatings. (**b**) % Transmission in the IR region at a wavelength of 2,000 nm as a function of temperature. As it can be seen, the plot resembles the MIT plot as obtained in Fig. 10(a) indicating the presence of MIT. (**c**) % Transmission in the IR region at a wavelength of 2,000 nm as a function of voltage applied. As seen from the image, a sharp drop in the % IR transmission can be bought about by application of voltage 12 V and higher with the lowest transmission occurring at 15 V.

The films grown in the S-2 configuration were tested for their potential to be used as a smart window coating by measuring their transmission spectra at different temperatures. As seen from Fig. 12(a), the transmission spectra of the films near IR region decreased with an increase in the temperature of the films. The transmission at 2000 nm reduced by more than 40% with rise in temperature by supplying 12 V or higher to the film. This reduction to the IR transmission with an increase in temperature confirm the potential of the multi-layered structure to be used as a smart window coating. Furthermore, the transmission data was plotted as a function of temperature and applied voltage. Figure 12(b) demonstrates the presence of a MIT, as seen from the transmission spectra with an increase in temperature. The information obtained from 12(b) is very similar to the electrical measurements done on the films as shown previously in Fig. 9(a). Furthermore, Fig. 12(c) shows the change in the transmission in the IR region as a function of voltage. As seen from the graph, by applying a voltage of 12 V and higher, the MIT in the films could be triggered, which leads to a decrease of the transmission in the IR region by over 40%. The films reach their lowest IR transmission by a voltage of ~15 V. Putting it in practical device terms, as the temperature of the films increases, by applying a voltage of ~15 V, the films can block off most of the IR rays entering the room and thereby keeping it cooler. In reverse, when the films are exposed to a colder temperature, even without application of any voltage, they can allow ~80% of the IR rays, which can help keeping the rooms warmer^7,42,43^.

## Conclusion

AZO and VO_2_ thin films grown in a novel multilayered structure using pulsed laser deposition were able to demonstrate a lower transition temperature and a narrow hysteresis width while maintaining a heating effect in the AZO films despite the severe differences in the growth parameters of both the films. Furthermore, the multi-layered structure revealed to be a potential candidate to be used in a smart window coating owing to its reduced transition temperature of <60 °C as compared to ~70 °C in bulk VO_2_, higher IR transmission at room temperature and below as well as a sharp drop in the IR transmission to a lower value of less than 30% upon application of a small voltage of ~15 V. The results obtained above determine the importance of strain in the films to tune the transition temperature of the films in the multi-layered structure. Furthermore, with the simplicity of the structure used and the potential of this structure to be used as a smart window device, it opens up doors to billions of dollars in energy savings which can then be used for the betterment of humanity^44,45^.

## Methods

### Deposition of VO_2_ thin films

VO_2_ thin films were deposited on c-0001 sapphire substrates by Neocera pulsed laser deposition using a KrF excimer laser (UV, 248 nm). The sapphire substrates were cleaned using an ultrasonic cleaner in solutions of acetone, isopropyl alcohol and de-ionized water for 10 minutes each. The substrates were blow dried using nitrogen (N_2_) after each step.

The VO_2_ thin films were grown from a V_2_O_5_ target with a laser energy of 220–240 mJ per pulse at a repetition rate of 5 Hz in a chamber with a base pressure of less than 5 × 10^−8^ Torr. The substrates were maintained at a temperature of 630 °C. The deposition was carried out in an oxygen partial pressure of 1 mTorr. The as-grown samples were then annealed at 660 °C for a duration of 1 hour in an oxygen pressure of 100 mTorr.

### Deposition of Al doped zinc oxide thin films

The Al doped zinc oxide (AZO) thin films of different thickness (90 nm, 165 nm and 220 nm) were deposited from a pressed AZO target. The deposition was carried out at a substrate temperature of 350 °C in vacuum (pressure <5 × 10^−8^ Torr) without the use of any process gas. The laser energy was set to 220–240 mJ/pulse with a repetition rate of 7 Hz. The oxygen deficient environment was used in order to create more oxygen vacancies in order to maximize the Joule heating effect of the films.

### Deposition of the multi-layered thin film

The multi-layered films were grown in two different configurations: one with VO_2_ films deposited on top of the AZO films (S-1) and the other with AZO films deposited on the VO_2_ films with the AZO films being deposited on three out of four edges of the VO_2_ films (S-2). Schematics of both the structures are depicted in Fig. 1(a,b), respectively.

### Characterization of the thin films

Structural characterization was performed on the films to determine their crystallinity using a Rigaku powder X-Ray diffractometer. The thickness of the films was measured using a Bruker Dektak profilometer. The films were characterized for their electrical properties to determine the dependence of resistance with temperature using a linear four-point probe configuration using a Keithley 6220 current source meter and a dual channel Keithley 2182 A nano-voltmeter which measured the voltage as well as the temperature of the films which was provided using a PID controlled oven. The data obtained from the linear four probe measurement was analyzed to determine the transition temperature of the films using MATLAB. The resistive heating effect on the films was measured using a FLIR 320 infra-red camera with the voltage being supplied using a HP E3633A DC power supply. The films were characterized to test for the transmission of UV, visible and near IR region using a Perkin Elmer Lambda 950 UV/Visible spectrometer. Voltage as well as temperature dependent transmission measurements were carried out on the films grown in the S-2 configuration, using the AZO layer to heat up the films.
